# Spread, Scale-up, and Sustainability of Video Consulting in Health Care: Systematic Review and Synthesis Guided by the NASSS Framework

**DOI:** 10.2196/23775

**Published:** 2021-01-26

**Authors:** Hannah M James, Chrysanthi Papoutsi, Joseph Wherton, Trisha Greenhalgh, Sara E Shaw

**Affiliations:** 1 Department of Knowledge Integration University of Waterloo Waterloo, ON Canada; 2 Nuffield Department of Primary Care Health Sciences University of Oxford Oxford United Kingdom

**Keywords:** delivery of health care, remote consultation, telemedicine, videoconferencing, spread and scale-up, sustainability, mobile phone, COVID-19, remote care, consultation, review

## Abstract

**Background:**

COVID-19 has thrust video consulting into the limelight, as health care practitioners worldwide shift to delivering care remotely. Evidence suggests that video consulting is acceptable, safe, and effective in selected conditions and settings. However, research to date has mostly focused on initial adoption, with limited consideration of how video consulting can be mainstreamed and sustained.

**Objective:**

This study sought to do the following: (1) review and synthesize reported opportunities, challenges, and lessons learned in the scale-up, spread, and sustainability of video consultations, and (2) identify transferable insights that can inform policy and practice.

**Methods:**

We identified papers through systematic searches in PubMed, CINAHL, and Web of Science. Included articles reported on synchronous, video-based consultations that had spread to more than one setting beyond an initial pilot or feasibility stage, and were published since 2010. We used the Nonadoption, Abandonment, and challenges to the Scale-up, Spread, and Sustainability (NASSS) framework to synthesize findings relating to 7 domains: an understanding of the health condition(s) for which video consultations were being used, the material properties of the technological platform and relevant peripherals, the value proposition for patients and developers, the role of the adopter system, organizational factors, wider macro-level considerations, and emergence over time.

**Results:**

We identified 13 papers describing 10 different video consultation services in 6 regions, covering the following: (1) video-to-home services, connecting providers directly to the patient; (2) hub-and-spoke models, connecting a provider at a central hub to a patient at a rural center; and (3) large-scale top-down evaluations scaled up or spread across a national health administration. Services covered rehabilitation, geriatrics, cancer surgery, diabetes, and mental health, as well as general specialist care and primary care. Potential enablers of spread and scale-up included embedded leadership and the presence of a telehealth champion, appropriate reimbursement mechanisms, user-friendly technology, pre-existing staff relationships, and adaptation (of technology and services) over time. Challenges tended to be related to service development, such as the absence of a long-term strategic plan, resistance to change, cost and reimbursement issues, and the technical experience of staff. There was limited articulation of the challenges to scale-up and spread of video consultations. This was combined with a lack of theorization, with papers tending to view spread and scale-up as the sum of multiple technical implementations, rather than theorizing the distinct processes required to achieve widespread adoption.

**Conclusions:**

There remains a significant lack of evidence that can support the spread and scale-up of video consulting. Given the recent pace of change due to COVID-19, a more definitive evidence base is urgently needed to support global efforts and match enthusiasm for extending use.

## Introduction

There is global interest in video consultation services, including Skype, Teams, FaceTime, and other Voice over Internet Protocol (VoIP) media, to facilitate synchronous patient-to-provider video communication in health care [1,2]. Studies have shown positive patient outcomes, reduced travel and costs, improved communication, decreased waiting times, and increased accessibility for patients [3]. Governments and health administrations view such technologies as a means of better managing demand and improving care [1]. The COVID-19 pandemic, and the requisite need for self-isolation and social distancing, has prompted rapid and widespread adoption of video consultations [4].

Evidence on the use of video consultations in health care is mixed. There is a rapidly growing literature on the feasibility, safety, acceptability, and effectiveness of video consultations across clinical areas [5] including diabetes [6,7], rehabilitation [8,9], mental health and addiction [10], cancer [11], palliative care [12,13], long-term care [14], geriatrics [15], postpartum support [16], and primary care [17]. Studies tend to be small scale and focus on initial adoption in a research context [2,5,18]; adopt a technology-centric approach (in which the technology is the primary focus, rather than the service or organization by which the technology is being used); and use trial methodology to study whether video consultation technology works or not. Despite calls for urgent action [19-22], this trend has continued into the COVID-19 pandemic. Little is currently known about how to successfully spread and scale up video consulting for sustained use across settings [23-25].

A small number of studies have explored the technological, contextual, and practical challenges to be overcome if video consulting is to become more widespread. One multilevel qualitative study, conducted in the English National Health Service (NHS) and undertaken by our team, examined the development, implementation, and use of video consultation services [2]. Focused on national-level policy, organizational-level implementation, and patient-clinician video consultations, the study identified a mismatch between the policy vision of video consultations replacing or supplementing a significant proportion of face-to-face care [26,27], and the substantial setup resources, ongoing human effort, and time needed to embed video consultations in routine care. Findings suggest that, even where there is significant policy impetus and demand [4], those implementing video consultation services face significant challenges in redesigning existing services and implementing new pathways.

Appreciation of the potential for longer term sustainability of this new service model is crucial in the context of the unfolding COVID-19 pandemic, which has brought a rapid need for alternatives to face-to-face contact and hence for the spread and scale-up of video consultations. We therefore conducted a systematic review of the opportunities and challenges to widespread implementation (what we refer to as “scale-up” or “spread”) of video consultation services in health care, asking the following questions:

1. What theoretical frameworks have been used in this literature and for what purpose?
2. What opportunities and challenges have been identified in the literature on the spread, scale-up, and sustainability of video consultations?
3. What transferable insights can be useful for policy and practice? What questions remain unanswered?

Our main concern is to identify and evaluate challenges to the scale-up and spread of video consultations from the existing research literature and, from this, to inform the rollout and longer term sustainability of video consulting and the research agenda that can support it. Spread, scale-up, and sustainability are often used as interchangeable terms without a standard definition or adequate theorization (see Multimedia Appendix 1). In this review, we do not adopt a single definition of spread, scale-up, and sustainability, as we are interested in surfacing the different ways in which studies on video consultations have employed and operationalized these terms.

## Methods

### Information Sources and Search Strategy

In December 2018, we systematically searched 3 databases: PubMed, CINAHL via EBSCOhost, and Web of Science. The search was updated in March 2020. Our search strategies, developed with the help of a research librarian, used a mix of keywords, Medical Subject Headings (MeSH), and Major Headings (MH), as provided in Multimedia Appendix 2. We identified search terms bottom-up by examining titles, keywords, and frequently used phrases in relevant literature. For example, we derived terms such as “telemedicine” and “remote consultation” from literature on virtual health care and terms like “scalability” and “spread” from implementation science articles. We used broad telehealth and telemedicine terms to be able to address the variability in the terminology both in the scale-up and spread literature and in the way video consultation services have been described. The lead author reviewed PROSPERO (International Prospective Register of Systematic Reviews) prior to the study to identify similar reviews, which informed the search strategy and review focus. We referenced our initial search strategy against two published systematic reviews on telegenetics [28] and implementation science [29] to identify supplementary terms. Filters were applied to limit the results to published peer-reviewed articles. We focused on published literature and did not search grey literature. PRISMA-P (Preferred Reporting Items for Systematic Review and Meta-Analysis Protocols) was used to draft the protocol for this review (unpublished) and PRISMA guidelines were consulted throughout the review as a guide.

### Eligibility Criteria

Inclusion criteria are summarized in Table 1. Included articles were peer-reviewed and reported on synchronous, video-supported consultations that had been scaled or spread to more than one setting beyond the initial pilot or feasibility stage (either within the same organization or to other organizations or geographic settings). The video consultation technology could be stand-alone, or part of a larger telehealth innovation (eg, a website). We focused on video consultation services connecting a patient to their medical provider(s) as opposed to those connecting providers to specialists. Articles exclusively reporting use, feasibility, acceptance, or pilot implementation with no evaluation of the implementation process were excluded. Video technologies like Skype have only recently come into use in health care [2,30], hence we restricted our search to articles published since 2010. Beyond these restrictions, we kept a broad interpretation of the patient-provider relationship, allowing video-to-home telehealth and hub-and-spoke consulting connecting a rural center to a specialist at a different hospital or institution. Our understanding of the quality of the articles was informed by using the Critical Appraisal Skills Programme (CASP) checklist for qualitative research and the Mixed Methods Appraisal Tool (MMAT) for mixed methods studies; however, we did not exclude articles on the basis of quality appraisal but took this into account in the interpretation of their findings (as reflected in the results section).

**Table 1:** Inclusion and exclusion criteria.

Criteria	Inclusion	Exclusion
Time period	2010 onwards	Before 2010
Language	English	Not English
Video consultation format	Synchronous and video-based conferencing OR digital health care technologies that include synchronous video conferencing.	Asynchronous, app-based, text-based, or website-based formats.
Actors	Patient and health care provider communication	Any other video consulting service
Context	Relevant technologies that have been scaled or spread to >1 setting in acute or primary care or where scale-up and spread is being actively pursued	Usability studies, feasibility trials, single-location implementation, pilot studies, or studies that had not been scaled or spread beyond one setting
Format	Peer-reviewed articles	Anything else (eg, conference proceedings, books, workshop papers)

### Screening

Search results were imported into Zotero. Duplicates were removed, while retaining information from each search and preventing false duplicates from being merged. Each record was first screened by title and abstract (initially by HJ, then verified by JW and SS). Remaining articles were screened by reading the full texts (HJ screening 100%, SS a 15% sample), with any disputes resolved through discussion and consensus with JW. At each stage, articles were eliminated if they did not meet the inclusion criteria (Table 1). Full-text screening was iterative and required slight refinement of our inclusion criteria. We clarified that the articles must place sufficient emphasis on video consulting (rather than mention it in passing) and that different terms could be used to describe processes relevant to widespread implementation, scale-up, and spread.

### Data Extraction and Synthesis

We extracted data into a Microsoft Excel (Microsoft Corp) spreadsheet. At a descriptive level, we extracted data on study location, setting, clinical focus, definition of telehealth, service model and type of technology, and research design (Multimedia Appendix 3). We extracted data on how each of the papers conceptualized opportunities and challenges for the spread, scale-up, and sustainability of virtual consultations. We also extracted data about the predominant theoretical framework adopted in each study, and connected these with the three theoretical lenses that typically characterize studies of the spread and scale-up of innovations: (1) implementation science (ie, the systematic and structured application of improvement techniques and frameworks), (2) complexity theory (ie, paying attention to unpredictability and interdependencies in complex systems), and (3) social science approaches (ie, emphasizing social, human, and material elements in large-scale change efforts) [31].

**Figure 1:**
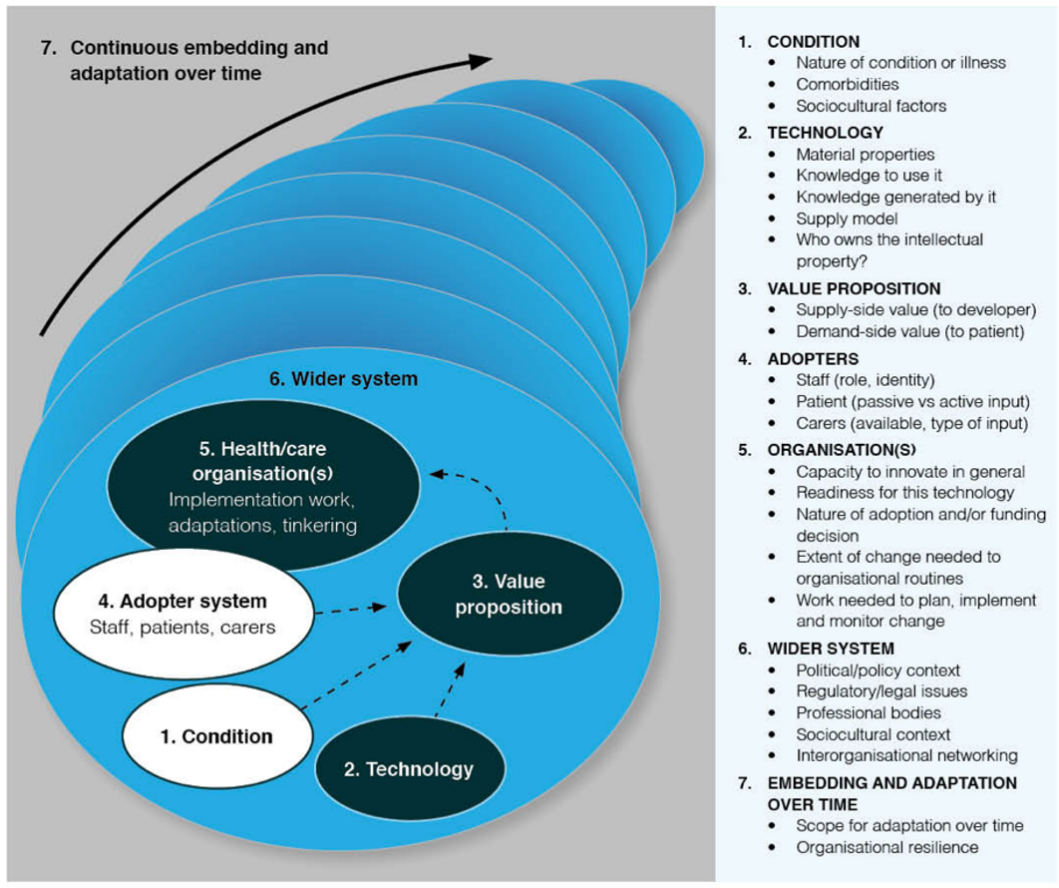
The NASSS framework for Nonadoption, Abandonment, and challenges to Spread, Scale-up, and Sustainability of health and care technologies.

We worked inductively to surface the challenges to and opportunities for the spread and scale-up of video consulting across studies. Sensitized by the Nonadoption, Abandonment, and challenges to the Scale-up, Spread, and Sustainability (NASSS) of health and care technologies framework [23], we then worked deductively to ensure we had not missed any of the 7 domains identified as relevant to the widespread adoption and nonadoption of innovations (ie, an understanding of the health condition[s] for which video consultations are being used, the material properties of the technological platform itself, the value proposition for patients and developers, the role of the adopter system, organizational factors, wider macro-level considerations, and emergence over time; see Figure 1).

We piloted and refined this process on one paper and then extended the process across the rest of our data set. Using content analysis, we compiled a descriptive overview of the opportunities and challenges identified in the papers, including frequency distributions. We then applied a more analytical lens to synthesize and interpret our findings using the NASSS framework [23].

## Results

### Description of Papers and Overview of Findings

**Figure 2:**
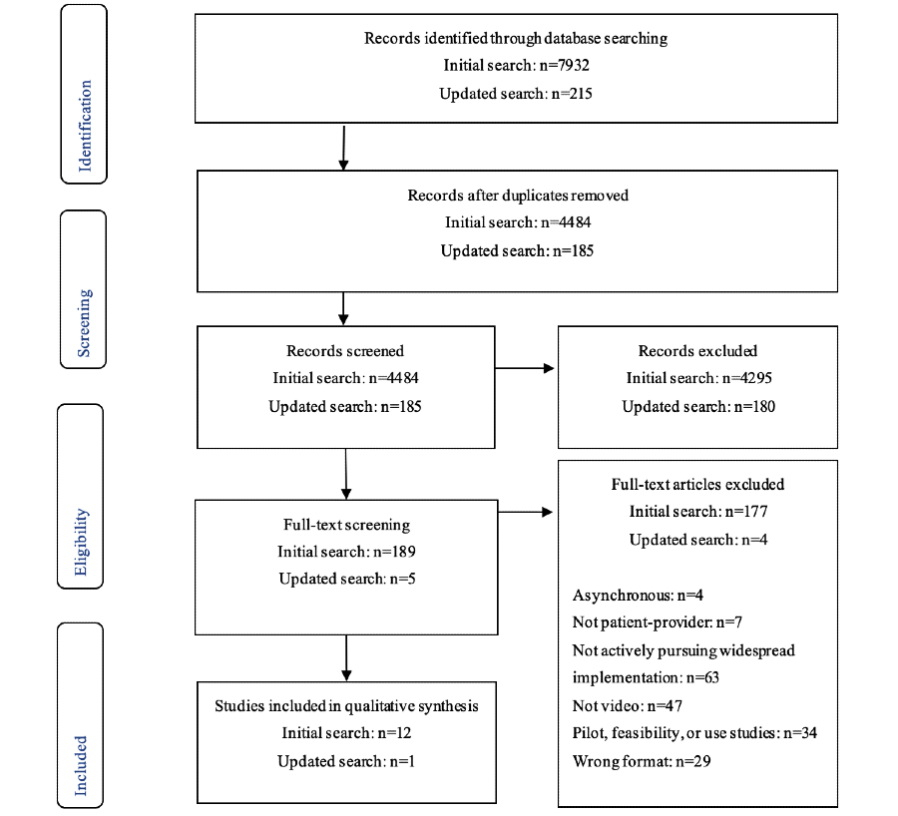
PRISMA flow diagram depicting search and screening processes. PRISMA: Preferred Reporting Items for Systematic Review and Meta-Analysis.

Initial searches identified 4484 unique articles published between January 2010 and December 2018, with 193 articles identified for full-text screening and 12 initially included in the final review. An updated search in March 2020 returned 185 new articles for screening, of which 5 were identified for full-text screening. One new relevant article was identified, bringing the total records included in the review to 13 (Figure 2).

Diverse research designs were employed in the included studies, including interpretive case studies [32], structured or semistructured qualitative interviews [33-35], mixed methods combining qualitative and quantitative data [2,36-38], action research and deliberative methods [39], prospective implementation studies including quantitative activity and performance indicators [40-42], and retrospective case analysis of systemwide use of video consultations [43] (Multimedia Appendix 3). A review of the included studies using the CASP and MMAT appraisal tools indicated that they generally fulfilled relevant quality standards, although in a few articles it was not clear how data collection or analysis methods were applied, sample sizes were small, or there was inadequate information on sampling frame; furthermore, they remained at a highly descriptive or even purely illustrative level of analysis.

There was ambiguity in the use and variations of the term telehealth, with three studies giving no definition and others defining telehealth variably depending on study focus (Multimedia Appendix 3). The terms telehealth, telemedicine, and telemental health were common and often used interchangeably: 11 articles (85%) used the terms “video telehealth,” “telehealth,” “telemedicine,” or “telecare,” while two used the term “video consultations” [2,38].

Papers described 10 different video consultation services in 6 regions: Australia [39], Cabo Verde [41], England [2,38], Nepal [32], Norway [36], and the United States (Multimedia Appendix 3). Studies described 3 types of video consultation services: (1) video-to-home services [2,34,38,39,42] that connected providers directly to a patient who received a video consultation on their own device; (2) hub-and-spoke models [32,33,35,37,40,41] that connected a provider at a central hub to a patient at a rural spoke center who called in with the assistance of a provider at that organization; and (3) large-scale top-down evaluations scaled up or spread across a single country [36] or a nationwide health administration [43]. Services covered various clinical specializations including rehabilitation [35,39], geriatrics [39], cancer surgery [2,38], diabetes [2,38], and mental health [34,37,40,42], in addition to general specialist care and primary care [32,33,36,41,43]. All studies presented empirical findings relating to services that had (to varying degrees) undergone, were undergoing, or were about to undergo scale-up or/and spread.

The different approaches and frameworks used in the papers reviewed reflect different logics of change. This has implications for the way spread, scale-up, and sustainability are operationalized, studied, and conveyed. Many of the articles included in the review emphasized a logic of change underpinned by implementation science (Multimedia Appendix 3). Bauer et al [37] used the Reach, Efficacy, Adoption, Implementation, and Maintenance (RE-AIM) framework in the quantitative arm of their evaluation on telehealth for bipolar disorder in the Veterans Affairs (VA) health care system to quantitatively assess the extent and patterns of implementation and sustainability. They also drew on the integrated Promoting Action of Research Implementation in Health Services (iPARIHS) framework to analyze qualitative data on challenges and opportunities for program implementation and sustainability. The Consolidated Framework for Implementation Research (CFIR; not designed specifically to look at implementation of software) was used in another implementation science–focused study to identify analytical constructs for collecting and analyzing provider perspectives on video telehealth in mental health services for US veterans [34,55]. Wade et al [39] used grounded theory to construct a process model of change management in large-scale home telehealth in South Australia, highlighting leadership support as a key facilitator. When reflecting on the transferability of their findings, this paper also makes reference to complex change and systems theory [39].

The above studies primarily report the use of frameworks as part of evaluating, rather than guiding, spread and scale-up efforts. Another two articles employed frameworks to support spread and scale-up efforts, although they did not draw on relevant approaches such as the Going to Full Scale framework, 3S infrastructure, or the Dynamic Sustainability framework [58-60]. In a prospective analysis in Cabo Verde, the “initiate-build-operate-transfer” approach was described as the basis for delivering a countrywide telemedicine network. This seemed akin to a phased implementation framework, although it was supplemented by a range of additional measures to address sustainability factors as reported in relevant literature, including careful training and capacity development [41]. In a second prospective study, the PARIHS framework, including use of external facilitation, was employed as a systematic approach to guide extensive implementation of psychotherapy for posttraumatic stress disorder (PTSD) by the US Department of Veterans Affairs [40].

Complexity was mentioned in a few articles, although not as a key organizing framework. Alami et al [36] analyzed data from their mixed methods study on national telemedicine implementation in Norway without employing a conceptual framework; however, their discussion emphasizes complexity, adaptive capability, and participatory approaches. Darkins [43] drew on diffusion of innovations theory to organize his analysis of telehealth expansion in the US Veterans Health Administration over a 10-year period, referring as well to the “complex adaptive environment” and “systems approach” taken to achieve spread.

Social science theory was also employed, although rarely in a highly theoretical mode. In their study on telemental health services for rural American Indian communities, Brooks et al [33] used diffusion of innovations theory to retrospectively examine factors that influenced widespread adoption. Martinez et al [35] applied a sociotechnical perspective in their study of health providers’ perspectives on video telehealth for US veterans with spinal cord injuries. This led to identification of social and technical factors that influenced telehealth implementation across care facilities. In their discussion, they also reflected on the interdependencies and relationships between the different sociotechnical aspects of the system, viewing health care teams as complex adaptive systems. Bhatta et al [32] presented a descriptive overview of opportunities and challenges for telemedicine in Nepal and included some, albeit limited, discussion of their findings from an information infrastructures perspective to highlight the importance of installed base. Finally, the VOCAL study took an explicit social science approach by theorizing video consultations using technology-enhanced Strong Structuration Theory, which assumes a dynamic and reciprocal link between the social environment, human interpretations and actions, and technologies [2,38].

### Reported Opportunities and Challenges

A total of 38 opportunities to scale-up, spread, and sustainability and 47 unique challenges were reported across the 13 articles. The most common opportunities included the availability of clinical and/or nonclinical telehealth champions or coordinators (n=8), provider acceptance (n=4), absence of billing or licensure restrictions (n=3), adequate funding (n=4), and strong interorganizational communications (n=4). The most common challenges were lack of technical telehealth-specific support for clinical staff (n=6), need for redevelopment of workflows and organizational routines (n=6), financial pressures (n=5), and lack of training (n=5).

Not all articles explicitly reported opportunities and challenges: 10 (77%) reported both challenges and opportunities [2,33-35,37-41,43], 2 (15%) reported challenges exclusively [32,36] and 1 (8%) article focused more on opportunities and how these could be translated to other settings [42].

### Challenges to Scale-up, Spread, and Sustainability

We synthesized the data extracted from the 13 articles according to the 7 domains of the NASSS framework [23] (Figure 1).

#### Domain 1: The Condition

This domain encompasses the clinical and sociocultural aspects of the health condition and associated comorbidities, acknowledging that not all individuals with the same condition would benefit equally from health technologies [23]. The complexity of the condition plays a role in determining the suitability of patients for video consultations and hence influences the potential for scale-up, spread, and sustainability [23]. In total, 7 of the studies reported the conditions for which video consultations were used, including dermatology, diabetes, antenatal diabetes, postoperative cancer, spinal cord injuries, bipolar disorder, PTSD, and other mental health conditions. Of these studies, 6 paid limited attention to how clinical characteristics played a role in the successful spread of video consulting services [32-35,37,42], although there was mention of provider concerns around using video consulting in specific conditions (eg, “for patients who are at high risk of suicide or who have psychotic symptoms”). This was mainly reported by participants with no previous experience of video consulting [34].

Opportunities for and challenges to the spread and scale-up of video consulting by type of clinical condition were reported in detail in only one study [2,38]. For example, video consulting in an antenatal diabetes clinic was abandoned given the involvement of multidisciplinary teams across departments, the duration and severity of the condition (short-term, high-risk), and the use of patient-held medical records that were unavailable to the clinician conducting the video consultation [2,38]. Preoperative cancer surgery was deemed too complex for video consulting given the necessity of a physical exam combined with there likely being no pre-existing relationship between the patient and clinical team, but postoperative follow-up within the same clinic was deemed more appropriate [2,38]. Other studies pointed to the difficulties in conducting physical examinations as constraining spread and scale-up. For example, an investigation of video consulting scale-up for spinal cord injury suggested that a “common concern was the perceived limits to evaluate physical symptoms over [video]” and the difficulty to assess clients’ complaints without being able to physically examine them [35]. One study noted that for patients with agoraphobia, the video medium removed the need to travel to the clinic and allowed patients to more easily receive mental health support [42].

#### Domain 2: The Technology

The technology domain encompasses the materials, data, knowledge, and supply features of the technology [23]. This domain focuses not only on the physical technology and knowledge required to use it, but also on how the technology shapes (and becomes shaped by) the potential for scale-up and spread [23]. The 13 papers referred to various video consultation programs but focused on their implementation process rather than providing details of the video conferencing technologies used. Some descriptions remained vague; for example, one article referred to a “simple technology that is only used for consultation purposes” [32]. Others provided a brief overview of changes in technological solutions, from early telehealth programs that used “commercial off-the-shelf videoconferencing systems, ones that had been developed for administrative, not clinical purposes” to latter stages using more sophisticated technological tools, such as teleretinal imaging [43]. Companion devices were also mentioned as providing opportunities for enhanced video consulting, including “an e-stethoscope, vital signs monitor, and dermatology camera, but also the e-electrocardiograph and ultrasound probe” [41]. Little emphasis was placed on describing the material features of the technologies; however, 6 studies reported technical challenges restricting spread, including the following: lack of reliability of the video conferencing technology including audio or video interruption/failure [2,32,34,35,38], inadequate maintenance [32], and unreliable internet accessibility or bandwidth [32,35,41].

Brooks et al [33] also reported on challenges setting up the infrastructure to be able to accommodate video consulting: “respondents noted many challenges in the clinic implementation process. Among these were … setting up the telehealth backbone.” In Norway, infrastructural differences between the different regions have challenged the spread of telemedicine and meant that the national eHealth strategy could not be implemented consistently [36]. Elsewhere, unreliable or inconsistent national internet capability and service was reported as a challenge to implementation [32,41]. For example, in Nepal, “due to the irregular supply of electricity and slow internet service, it is difficult … during video conference consultations due to frequent disconnection, blurry images, and unclear sound” [32]. Additionally, widespread implementation requires patients to have access to the appropriate “infrastructure” to participate in a video consultation (computer, webcam, data allowance) [40].

Across all papers reviewed, available technologies for video consultations posed challenges for establishing remote models of care as well as spread and scale-up. Authors reported that support and high-quality training were necessary to achieve widespread growth [2,32-43]. This was illustrated strongly in a large-scale implementation in Nepal: “Another challenge was related with lack of competence and training among the personnel involved in the rural-telemedicine program” [32]. Even when specialist information technology (IT) staff were available, a lack of service and procurement standards hindered spread across the system, as in the example of video consulting in the Veterans Health Administration: “telehealth projects were developed using bespoke interfaces at an individual medical center, ones that were not replicable across the system. Both Veterans Health Administration (VHA) IT and biomedical engineering supported telehealth but without consistent standards for equipment purchase, installation, service, warranty, and help-desk arrangements” [43].

#### Domain 3: The Value Proposition

The value proposition domain is concerned with whether or not the technology is worth developing or introducing for clinicians, patients, and suppliers [23]. It can influence upstream supply of the technology and uptake and desirability on the demand side, impacting scale-up and spread [23]. The majority of papers (n=10) discussed the value proposition for video consulting and how this was articulated by different program stakeholders. Value proposition was primarily understood in terms of clinical stakeholder perception of patient demand and interest [39,42], perceived clinical need/utility [2,32,33,35,38,41,43], and supplier benefit [2,38]. For example, in an evaluation of video consulting for individuals with bipolar disorder, growth was enabled by technology that was “successfully filling a need perceived by providers” [37]. In another study, Lindsay et al [42] reported that the “natural disaster Hurricane Harvey offered unique motivation for previously reluctant providers to use VTH delivery to connect with their patients during the crisis and beyond,” not only emphasizing the value proposition changing mid-implementation, but also enabling rapid scale-up in ways not previously imaginable.

A weak or poorly articulated value proposition posed challenges for scale-up and spread (n=2). For example, Martinez et al [35] reported that “some providers encountered initial hesitance from individuals with Spinal Cord Injuries or Disorder which they mostly attributed to patients’ uncertain feelings about the new technology.” Interian et al [34] also described how a video consulting program aimed at improving access to mental health care did not spread successfully in an urban environment compared to other settings, because providers did not perceive a local need for the technology: “A second issue involving provider buy-in pertained to the perceived local need for implementation.”

The upstream supply of technology (ie, the relationship between the organization and the supplier or developer) was reported as a facilitator by Greenhalgh et al [38], who explained that scale-up in a Skype-based service was facilitated partially by “clear benefit for both the technology supplier and the patient.” Contrastingly, Bhatta et al [32] reported how uncertain supplier relationships and funding threatened the sustainability and scale-up of their program in Nepal.

#### Domain 4: The Adopter System

The adopter system considers the staff, patients, and caregivers and their potential and desire to adopt and to continue to use a technology [23]. Staff concerns with video technologies were reported in all papers (n=13) and included the following: lack of provider buy-in and resistance to change [2,33,36,38,39,42]; lack of adequate training and telehealth staff to support sustainable operation of the video consulting programs [32,34,35,37,41], and the mobility of human resources within and between systems including “unplanned transfer of healthcare workers” [32]; the “high turnover of clinicians” [36], and availability of staff, space, and equipment [37]. Providers were slow or reluctant to buy in because they did not trust the technology or consider it clinically applicable [33,34,42]; did not have experience with the technology [34,40]; did not have adequate time to devote to the new technology [2,38]; did not have enough evidence to support video consulting use, resulting in resistance to changes in care models, as they found existing care models to adequately fulfill patient needs [39,40]; or lacked training and technological literacy or ongoing support [32,35].

Spread and scale-up were enabled by an adopter system with engaged and committed staff who were given protected time by the organization to conduct video consultations, as mentioned by Interian et al [34]: “having a team of providers who were detailed solely to provide mental health services by using telehealth technology and had protected time to do so… was rated as having a strong positive impact on V2H [video-to-home telehealth] implementation.” Engaged leadership staff were also reported to be pivotal for promoting uptake and creating conditions for sustained and widespread provider uptake [42].

Challenges to and opportunities for widespread implementation for patients and caregivers were reported in 6 articles [2,32-34,38,42]. Reported patient and caregiver challenges to and opportunities for widespread implementation included patient trust and acceptance of video consulting [2,33,38], the patient’s ability to transport themselves to a “hub” site (for hub-and-spoke video consulting [33]), and patients requiring appropriate technology to participate in video consultations [34]. In Nepal, challenges for patients were numerous and included cultural barriers (eg, patients’ feelings of inferiority to health care workers; women and older patients not speaking for themselves; lack of confidence) and literacy concerns, which were reported to make “it difficult and time consuming to use [video consults] for such patients” [32]. When patients were in front of a screen, it was reported by staff that patients felt uncomfortable participating in the consultation. For mental health care in the United States, patients reported liking the convenience and privacy of video consulting; however, these responses were collected from patients with repeated prior use of video consulting services, possibly excluding important challenges of patients with little or no experience with video consultations [42].

Despite the abundance of challenges to and opportunities for widespread implementation reported from the provider perspective, there was little in-depth consideration of the impact of patient and caregiver adoption on widespread implementation and how the work and acceptance of these groups may have influenced widespread and sustainable implementation. Of the articles that did report patient or caregiver challenges [2,32-34,38,42], these were primarily clinician perceptions, rather than direct reports from patients or caregivers.

#### Domain 5: The Organization

The organization domain considers the capacity of the organization to innovate, its readiness for change, the nature of the funding decision, the extent of change in routines, and the work needed to implement change in the organization as it relates to the new technology [23]. These elements are crucial in scale-up, spread, and sustainability because they address how the organization might respond to emerging challenges and how the innovation coevolves with organizational structures and processes [23]. Challenges reported in 5 articles included the labor-intensive process of scheduling video consultation appointments within and across organizations and the need to redesign organizational processes around the technology [2,36-38,40]. As Bauer et al [37] reported: “The scheduling process was noted to be labor-intensive, requiring a three-way match among the veteran’s schedule, the consultant’s schedule, and telehealth room availability.” Other challenges included how sites with existing video consulting programs and pre-existing routines may be hesitant or slower to implement new technologies and redesign workflows around a new technology [40] and how embedding video consultations in a clinic “involved significant reworking of … processes in ways that took account of the ‘virtual’ presence of the patient” [38]. In some cases, redesign of organizational processes was prohibitive to implementation. For instance, lack of widespread video consulting implementation in Norway was at least partially attributed to the lack of organizational preparedness required to “integrate changes and initiate restructuring” [36].

Complexity in the organizational domain was managed more effectively when telehealth champions were available, as reported in 11 of the reviewed articles [2,33-37,39-43]. In the words of one author, telehealth champions were “vital to [the] growth of telehealth” [43]. Telehealth champions were typically clinical staff [2,34,36,41-43]. Other roles included health care coordinators or IT staff [33,35,40,43].

#### Domain 6: The Wider Context

The wider context domain considers the political, regulatory, professional, and sociocultural aspects of video consulting implementation [23]. These factors shape the context in which the technology is implemented and influence the potential for growth and sustainability of video consulting services. At least one challenge or opportunity related to political, regulatory, cultural, contextual, or financial factors was reported in all studies (n=13) [2,32-43]. Political challenges included government instability, which affected related policy development, as a study in Nepal explained: “Due to the unstable political situation and frequent change of government, the policy related with the health care delivery system is fragile” [32]. By contrast, in the island country of Cabo Verde in Western Africa, there was a high-level political mandate for video consulting and a favorable collaboration environment with clear responsibilities between stakeholders, including international nongovernmental organizations. This facilitated the nationwide scale-up and spread of video consulting: “…the collaboration among the … government, the donor, and the implementing agency is superb, with clear terms of references for each of the players” [41].

Even when the political environment was more stable, papers reported that national strategies did not always emphasize the potential for scale-up and spread against other priorities. Take the following report from an evaluation in Norway, which suggests that national approaches “were mainly focused on messaging services and electronic exchange, while less attention was paid towards telemedicine services, especially videoconferencing” [36]. There was a gap in the papers reviewed, on the relevance of interorganizational networking for supporting and sustaining the spread and scale-up of video consulting [24]. Brooks et al [33] highlighted the importance of information (eg, background information about services, clinical protocols) from other organizations using telehealth in promoting take-up and spread. Two other papers reinforced this [2,43], referring to, for instance, “telehealth communities,” “collaborations across clinics and staff,” and “collaborations with…government partners” not only as important catalysts for change, but also as critical to the ongoing evolution and spread of services. None of the papers mentioned specific interorganizational initiatives (eg, quality improvement collaboratives). This was a surprising silence given the recognized importance of interorganizational networking for the spread of innovation [44,55,61].

Absence of or ambiguity in reimbursement was another common contextual barrier that influenced wider spread of video consultations [36]. In a UK study, the authors reported that, although lack of clarity on remuneration was often raised as a key barrier by implementing teams, published policy documents rarely explained how “reimbursement for virtual consultations would be implemented” [38]. In contrast, centralized funding for video consulting in the Veterans Health Administration in the United States meant that the “availability of national support infrastructure and the absence of billing for services were distinct advantages” [37].

#### Domain 7: Interactions Between Domains and Adaptation Over Time

The final domain, embedding and adaptation over time, focuses on the scope for adaptation and the resilience of the organization in the face of implementation and potential spread and scale-up [23]. One study explicitly recognized interactions between domains [2,38] by looking across three levels of data relating to spread and scale-up of video consulting: micro (individual users), meso (organizational processes and systems), and macro (national policy and wider context) [2,38]. They reported how challenges such as the condition interact with the organization, determining the ability to scale up video consulting services. For example, in a scale-up of 4 video consulting services, the challenges and opportunities for each service were connected across the domains; simply assessing a patient as having a theoretically appropriate condition for video consulting (micro) and having a supportive national policy (macro) did not guarantee success. Their specific interacting challenges included financial challenges, organizational structure, technical challenges, and the existing structure of treatment [2,38].

Factors impacting resilience, “the intrinsic ability of a system to adjust its functioning prior to, during, or following changes and disturbances so that it can sustain required operations, even after a major mishap or in the presence of continuous stress” [62], and adaptation over time were reported in three articles [32,42,43]. Notably, the absence of sustainable financial models was reported to threaten sustainability [32]. However, in a comprehensive description of the 20-year evolution and adaptation of telehealth within the VHA in the United States, Darkins [43] illustrates a dynamic transition from a fragmented health care system with many short-term pilot telehealth implementations that slowly scaled up and increased the scope of video consulting services and the associated infrastructure [43]. Lindsay et al [42] provided detailed resolution of adaptation over time for one health center within the VHA. In this implementation project, resiliency and sustainability were enabled by introducing video consulting into general practice rather than training specific providers and using a flexible implementation approach that can be modified for different health system contexts. Furthermore, the authors emphasized how measuring and assessing outcomes of implementation and sustainability then sharing those results with stakeholders “increases motivation and momentum for practice change and enables site to respond to challenges in real time” [42].

## Discussion

### Principal Results

This systematic review contributes valuable insights about the potential for scale-up, spread, and sustainability of video consulting and a novel interpretation through the application of the NASSS framework. A key finding is the paucity of evidence thoughtfully articulating challenges to the scale-up and spread of video consulting, which sits uncomfortably alongside the current global enthusiasm for expanding the use of video consulting services. The review also reveals an absence of concrete operationalization and theorization of scale-up and spread, exemplified by the absence of analysis and concrete definitions and terminology, as well as a lack of appreciation of complexity (particularly, but not only, in relation to the clinical condition and organizational implementation). The articles view spread and scale-up as the sum of multiple implementations, rather than theorizing the distinct processes required to achieve widespread adoption.

Findings from the review pointed to potential enablers of the spread and scale-up of video consulting services, notably regarding the influence of the presence of a telehealth champion, especially during initial spread [63], as well as leadership at multiple levels, appropriate reimbursement mechanisms, user-friendly technology, pre-existing relationships between staff [64], and adaptation (of technology and services) over time [65]. Findings also raised a number of challenges including the following: technically challenged staff, resistance to change, cost, reimbursement, and patient characteristics [24], as well as project management, patient recruitment, leadership involvement, funding, absence of a long-term strategic plan, resistance to change, workflow changes, lack of resources, and liability [64-66]. These challenges are consistent with previous research concerning small-scale implementation and diffusion of video consulting services and the scale-up and spread of other eHealth interventions [67,68]. They also indicate that the same things that interfere with implementation at one site [eg, 48] are also seen as interfering with spread and scale-up [5,7,17,24], although this may be an artifact of how spread is commonly studied as the sum of multiple implementations.

There are gaps in the existing evidence on the spread and scale-up of video consulting. This was particularly the case in relation to interorganizational networking, which is critical in enabling and sustaining innovation [24,30,64], and yet is not explored in-depth. There is also limited data on the material properties and design of different video consulting platforms, and on the value proposition in relation to spread and scale-up when a technology is free (eg, part of a research study) in the place where it was initially developed or implemented, but potentially costly in other settings.

As patients, health care organizations, and nations continue to look toward video consultations, it is essential to continue to theorize this domain. The absence of consistent theorization was demonstrated in this review by the absence of consideration of how challenges and opportunities might interact with each other and influence the process. One proposal to address this oversight is the theorization of scale-up and spread as social processes [69], prioritizing the interactions between actors and context to better inform scale-up or spread. Our review specifically draws on the NASSS framework to contribute a different way of viewing the spread and scale-up of video consultations, understanding opportunities and challenges as emergent, in constant tension, and inherently social. In conditions of complexity, spread and scale-up efforts may be further supported through the facilitation of interdependencies and relational aspects of change, processes that allow sense-making and experimentation, and scope for local adaptations and self-organization [31,70].

The COVID-19 pandemic is producing what is essentially a “natural experiment” as alternatives to traditional face-to-face consultations become a necessity to prevent viral transmission [4]. Interestingly, albeit much more localized, Hurricane Harvey was reported by Lindsay et al [42] to be an opportunity for provider buy-in for video-mediated mental health services. This suggests that the wider context of the COVID-19 pandemic might similarly enable widespread adoption of video consulting services.

### Limitations

This review is limited by likely bias within the articles, bias inherent in the reviewing process, and the lack of theorization in the included studies. Publication bias is a long-standing and recognized phenomenon in health research [71] and likely impacts the broadly positive results across studies included in this review. Given the lack of theorization in the included studies, it is hard to know how broadly the factors that may have acted as challenges or opportunities were considered in the evaluations. The consequence of this is that we cannot claim that this evidence provides a conclusive list of the challenges to scale-up and spread generally, nor even specifically to the studies included in this review.

The strengths of this review include the comprehensive searching methods used to identify a breadth of published research (albeit limited in numbers and scope). The analysis and synthesis of the listed challenges has also been framed by a comprehensive theoretical framework.

### Comparison With Prior Work

Our review contributes an important synthesis of knowledge about the spread, scale-up, and sustainability of video consulting. Our analysis, guided by the NASSS framework, has enabled us to examine a diverse body of evidence on video consulting services that have scaled or spread to more than one setting. This addresses a fundamental gap in the literature, with prior studies typically focusing on individual technologies or services, rather than organizational implementation and spread across settings [5].

To date, limited attention has been paid to the spread and scale-up of video consulting. Previous systematic reviews have tended to isolate areas of specific interest, focusing on selected aspects of video consulting, such as patient satisfaction [3,72], feasibility [5], clinical effectiveness and cost-effectiveness [73], and specialized clinical areas (eg, cardiology [74], diabetes [75], mental health and addiction [10], teledermatology [76]). Additionally, reviews have focused on telemedicine or eHealth in general without focusing on video consulting [24,77], with which our findings are consistent. Prior reviews have also focused exclusively on the technical aspects of video consulting technology [7]; considered technologies at any stage of implementation, rather than technologies with widespread adoption [24]; or restricted the types of articles included in the review to randomized control trials [78]. Studies have drawn on specific implementation frameworks to support and theorize spread and scale-up of video consulting, with a mechanistic logic of change being dominant. Other frameworks do exist that might support spread and scale-up (see [24] for an overview) but they have yet to be taken up in the context of expanding video consulting.

The studies we reviewed are broadly positive about video consulting. However, the small sample sizes, select nature of samples, and high losses to follow-up call into question any unqualified conclusion that video consulting is “effective.” The trials that have been undertaken on video consulting have provided few or no data on the organizational complexities of implementing a technology-based service, and do not address the question of how video consultation services become embedded in real-world settings.

To date, there have been only a handful of rigorous and theoretically grounded qualitative or mixed methods studies that explore the emergence of video consultation services naturalistically. Such studies have yet to systematically study spread and scale-up beyond the initial implementation.

### Conclusions

This systematic review identified opportunities and challenges for the scale-up and spread of video consulting. The application of the NASSS framework surfaced complexity in a number of domains, notably characterizing the importance of organizational support and the wider system in the scale-up and spread of video consulting services. Many articles viewed spread and scale-up purely as the sum of multiple implementations, rather than explicating and theorizing the distinct (social, interorganizational, and policy-related) processes required to achieve widespread adoption.

Given the recent impetus to implement video consulting services at pace and scale due to the COVID-19 pandemic, a more definitive evidence base is urgently needed to support global efforts, and match policy enthusiasm for the widespread use of these technologies. We strongly encourage decision makers and researchers to embrace relevant theoretical lenses that can aid spread and scale-up and ensure the future sustainability of what looks set to be a significant part of future health care delivery.

## Appendix

Multimedia Appendix 1 Spread, scale-up, and sustainability definitions: an overview.

Multimedia Appendix 2 Search terms used.

Multimedia Appendix 3 Summary of papers included in the review.

## Abbreviations

CVT: clinical video technology
CFIR: Consolidated Framework for Implementation Research
IBOT: initiate-build-operate-transfer
i-PARIHS: integrated Promoting Action on Research Implementation in Health Services
IT: information technology
MeSH: Medical Subject Headings
MH: Major Headings
NASSS: Nonadoption, Abandonment, and challenges to the Scale-up, Spread, and Sustainability
PARIHS: Promoting Action on Research Implementation in Health Services
PRISMA-P: Preferred Reporting Items for Systematic Review and Meta-Analysis Protocols
PROSPERO: International Prospective Register of Systematic Reviews
PTSD: posttraumatic stress disorder
RE-AIM: Reach, Efficacy, Adoption, Implementation, and Maintenance
VA: Veterans Affairs
VHA: Veterans Health Administration
VoIP: Voice over Internet Protocol
VTH: video-to-home
