# Supplementary material for: Spread, Scale-up, and Sustainability of Video Consulting in Health Care: Systematic Review and Synthesis Guided by the NASSS Framework
Source: J Med Internet Res. 2021 Jan 26;23(1):e23775. doi: 10.2196/23775 (PMC7837451; doi:10.2196/23775)
Supplement: Multimedia Appendix 1 [file jmir_v23i1e23775_app1.docx]

### Multimedia Appendix 1

### Spread, scale up and sustainability definitions: an overview.

A growing body of literature examines widespread implementation, scale-up and spread across diverse fields such as innovation studies, health services research and organizational science. Spread, scale-up, and sustainability are often used as interchangeable terms without a standard definition or adequate theorisation [1,2], and to variably describe dissemination, diffusion, implementation, and lifespan of technologies. Terms have often been employed synonymously or presented without a clear definition [3,4]. In some cases entirely different terms have been proposed to describe similar concepts [5].

Definitions of *scale-up* typically describe increasing coverage of a given service to more individuals [1,3,4,6–8]. Yet, within this common definition there are contradictions. For instance, geographic and organizational range in which this increased coverage occurs is sometimes described as *within* a geographic area [1,6]; at other times *beyond* a single geographic area and involving national or international implementation [3,4]; and sometimes not referenced at all [7,8]. Scale-up is also referred to as vertical diffusion, planned in a top down manner to expand use of innovations across a whole system [4,9], which is coherent with the definition of either moving from local to national use, or expanding within a health service. Others define scale-up as the process of establishing the infrastructure needed to support widespread implementation of innovations [10].

*Spread* is used less frequently, but is likewise inconsistently defined. According to some, the concept of *spread* captures the expansion of innovations beyond a single setting or locality to other settings or organizational contexts [1,7,11]. Horizontal diffusion, or the spread to other regions, is also used to describe intentional spread across different settings or along a care pathway [3,4,9].

*Sustainability* has been defined more consistently to denote the continued use of technologies over time. For example, Greenhalgh et al. [12] define sustainability as “*making an innovation routine until it reaches obsolescence”* [p582]. This definition is reflected in other publications. For example, in a review of the spread and sustainability of organisational changes Buchanan et al. [11] define sustainability as the “*process through which new working methods, performance enhancements, and continuous improvements are maintained for a period appropriate to a given context*” (p xxii-xxiii). In sum sustainability is typically thought of as something that happens organically and through a process of mutual adaptation.

As set out in the main paper, we do not adopt a single definition of spread, scale up and sustainability in our review of the literature, as we are interested in surfacing the different ways in which studies on video consultations have employed and operationalised these terms.

1. Shaw J, Shaw S, Wherton J, Hughes G, Greenhalgh T. Studying Scale-Up and Spread as Social Practice: Theoretical Introduction and Empirical Case Study. J Med Internet Res 2017 Jul 7;19(7):e244. PMID:28687532

2. Greenhalgh T, Robert G, Bate P, Macfarlane F, Kyriakidou O, Donaldson SL. Diffusion of Innovations in Health Service Organisations: A Systematic Literature Review. 1 edition. Malden, Mass: Blackwell Publishing Ltd; 2007. ISBN:978-0-7279-1869-7

3. Hanson K, Cleary S, Schneider H, Tantivess S, Gilson L. Scaling up health policies and services in low- and middle-income settings. BMC Health Serv Res 2010 Jul 2;10(Suppl 1):I1. PMID:20594366

4. Ilott I, Gerrish K, Pownall S, Eltringham S, Booth A. Exploring scale-up, spread, and sustainability: an instrumental case study tracing an innovation to enhance dysphagia care. Implementation Science 2013 Oct 29;8(1):128. [doi: 10.1186/1748-5908-8-128]

5. Nicolini D. Stretching out and expanding work practices in time and space: The case of telemedicine. Human Relations 2007 Jun 1;60(6):889–920. [doi: 10.1177/0018726707080080]

6. Greenhalgh T, Wherton J, Papoutsi C, Lynch J, Hughes G, A’Court C, Hinder S, Fahy N, Procter R, Shaw S. Beyond Adoption: A New Framework for Theorizing and Evaluating Nonadoption, Abandonment, and Challenges to the Scale-Up, Spread, and Sustainability of Health and Care Technologies. JOURNAL OF MEDICAL INTERNET RESEARCH 2017 Nov;19(11). [doi: 10.2196/jmir.8775]

7. Ly O, Sibbald SL, Verma JY, Rocker GM. Exploring role clarity in interorganizational spread and scale-up initiatives: the ‘INSPIRED’ COPD collaborative. BMC Health Services Research 2018 Sep 3;18(1):680. [doi: 10.1186/s12913-018-3474-2]

8. Eaton J, McCay L, Semrau M, Chatterjee S, Baingana F, Araya R, Ntulo C, Thornicroft G, Saxena S. Scale up of services for mental health in low-income and middle-income countries. The Lancet 2011 Oct 29;378(9802):1592–1603. [doi: 10.1016/S0140-6736(11)60891-X]

9. Charif AB, Zomahoun HTV, LeBlanc A, Langlois L, Wolfenden L, Yoong SL, Williams CM, Lépine R, Légaré F. Effective strategies for scaling up evidence-based practices in primary care: a systematic review. Implement Sci 2017 Nov 22;12(1):139. PMID:29166911

10. Greenhalgh T, Papoutsi C. Spreading and scaling up innovation and improvement. BMJ 2019 10;365:l2068. PMID:31076440

11. Buchanan DA, Fitzgerald L, Ketley D. The sustainability and spread of organizational change: modernizing healthcare [Internet]. Routledge; 2007 [cited 2018 Oct 30]. Available from: https://www.dora.dmu.ac.uk/xmlui/handle/2086/1635ISBN:978-0-415-37095-0

12. Greenhalgh T, Robert G, Macfarlane F, Bate P, Kyriakidou O. Diffusion of Innovations in Service Organizations: Systematic Review and Recommendations. The Milbank Quarterly 2004 Dec 1;82(4):581–629. [doi: 10.1111/j.0887-378X.2004.00325.x]
