# Supplementary material for: Spread, Scale-up, and Sustainability of Video Consulting in Health Care: Systematic Review and Synthesis Guided by the NASSS Framework
Source: J Med Internet Res. 2021 Jan 26;23(1):e23775. doi: 10.2196/23775 (PMC7837451; doi:10.2196/23775)
Supplement: Multimedia Appendix 2 [file jmir_v23i1e23775_app2.docx]

## Multimedia Appendix 2 – Search terms used.

**MAIN SEARCH**

PubMed:

| Bottom-up | 1.(scale[Tiab] AND (bring*[tiab] OR brought[tiab] OR taking[tiab] OR take*[tiab] OR increas*[tiab] OR going[tiab] OR implement[tiab])) OR (spread[Tiab] AND (innovation*[tiab] OR intervention*[tiab] OR technolog*[tiab] OR practice[tiab] OR care[tiab] )) OR Scale up[tiab] OR Scale-up[tiab] OR Scaling up[tiab] OR Scalability[tiab] OR implementation[tiab])  AND  2.(Telemedicine[mesh] OR Remote Consultation[mesh] OR Telehealth*[Tiab] OR Tele-health*[Tiab] OR telemed*[Tiab] OR Tele-med*[Tiab] OR eHealth*[Tiab] OR e-health*[Tiab] OR electronic consult*[Tiab] OR econsult*[Tiab] OR remote consult*[Tiab] OR virtual consult* [tiab]))  AND  3.(video[tw] or videoconference*[tw] or Skype[Tiab] or web-camera[tiab] or webcam [tiab] or Facetime[tiab] or web-cam[tiab])  AND  4. "2010/01/01"[PDat] : "2019/01/01"[PDat]) |
| --- | --- |

CINAHL

| Bottom-up | 1.((TI scale OR AB scale AND (TI bring* OR AB bring* OR TI brought OR AB brought OR TI taking OR AB taking OR TI increas* OR AB increas* OR TI going OR AB going OR TI implement OR AB implement)) OR (TI spread or AB spread AND (TI innovation* OR AB innovation* OR TI intervention* or AB intervention* OR TI technolog* or AB technolog* OR TI practice or AB practice OR TI care or AB care)) OR TI scale up AB scale-up OR TI scaling up or AB scaling up OR TI scalability or AB scalability OR TI implementation or AB implementation)  AND  2.(MH "telemedicine+"  OR TI telehealth* or AB telehealth*  OR TI tele-health* or AB tele-health*  OR TI telemed* or AB telemed*  OR TI tele-med* or AB tele-med*  OR TI ehealth* or AB ehealth*  OR TI e-health* or AB e-health*  OR TI electronic consult* or AB electronic consult*  OR TI econsult* or AB econsult*  OR TI remote consult* or AB remote consult*  OR TI Virtual Consult* or AB Virtual Consult*)  AND  3.(TX video  OR TX video conference  OR TI Skype OR AB Skype  OR TI web-camera OR AB web-camera  OR TI webcam OR AB webcam  OR TI web-cam OR AB web-cam  OR TI Facetime OR AB Facetime)  AND  4. EM 201001- |
| --- | --- |

Web of Science

| Bottom-up | 1. (((TI=scale OR TS=scale AND (TI=bring* OR TS=bring* OR TI=brought OR TS=brought OR TI=taking OR TS=taking OR TI=increas* OR TS=increas* OR TI=going OR TS=going OR TI=implement OR TS=implement)) OR (TI=spread OR TS=spread AND (TI=innovation* OR TS=innovation* OR TI=intervention* or TS=intervention* OR TI=technolog* OR TS=technolog* OR TI=practice OR TS=practice OR TI=care OR TS=care)) OR TI=scale up OR TS=scale-up OR TI=scaling up OR TS=scaling up OR TI=scalability OR TS=scalability OR TI=implementation OR TS=implementation)  AND  2. (TI=telehealth* OR TS=telehealth* OR TI=tele-health* OR TS=tele-health* OR TI=telemed* OR TS=telemed* OR TI=tele-med* OR TS=tele-med* OR TI=ehealth* OR TS=ehealth* OR TI=e-health* OR TS=e-health* OR TI=electronic consult* OR TS=electronic consult* OR TI=econsult* OR TS=econsult* OR TI=remote consult* OR TS=remote consult* OR TI=Virtual Consult* OR TS=Virtual Consult*)  AND  3. (TS=video OR TS=video conference OR TI=Skype OR TS=Skype OR TI=web-camera OR TS=web-camera OR TI=webcam OR TS=webcam OR TI=web-cam OR TS=web-cam OR TI=Facetime OR TS=Facetime)  AND  4.PY=(2010-2018))  AND  5.LANGUAGE: (English) |
| --- | --- |

**MAIN SEARCH UPDATED (NEW DATES)**

PubMed:

| Bottom-up | 1.(scale[Tiab] AND (bring*[tiab] OR brought[tiab] OR taking[tiab] OR take*[tiab] OR increas*[tiab] OR going[tiab] OR implement[tiab])) OR (spread[Tiab] AND (innovation*[tiab] OR intervention*[tiab] OR technolog*[tiab] OR practice[tiab] OR care[tiab] )) OR Scale up[tiab] OR Scale-up[tiab] OR Scaling up[tiab] OR Scalability[tiab] OR implementation[tiab])  AND  2.(Telemedicine[mesh] OR Remote Consultation[mesh] OR Telehealth*[Tiab] OR Tele-health*[Tiab] OR telemed*[Tiab] OR Tele-med*[Tiab] OR eHealth*[Tiab] OR e-health*[Tiab] OR electronic consult*[Tiab] OR econsult*[Tiab] OR remote consult*[Tiab] OR virtual consult* [tiab]))  AND  3.(video[tw] or videoconference*[tw] or Skype[Tiab] or web-camera[tiab] or webcam [tiab] or Facetime[tiab] or web-cam[tiab])  AND  4. "2019/01/01"[PDat]) : “2020/03/30"[PDat] |
| --- | --- |

CINAHL

| Bottom-up | 1.((TI scale OR AB scale AND (TI bring* OR AB bring* OR TI brought OR AB brought OR TI taking OR AB taking OR TI increas* OR AB increas* OR TI going OR AB going OR TI implement OR AB implement)) OR (TI spread or AB spread AND (TI innovation* OR AB innovation* OR TI intervention* or AB intervention* OR TI technolog* or AB technolog* OR TI practice or AB practice OR TI care or AB care)) OR TI scale up AB scale-up OR TI scaling up or AB scaling up OR TI scalability or AB scalability OR TI implementation or AB implementation)  AND  2.(MH "telemedicine+"  OR TI telehealth* or AB telehealth*  OR TI tele-health* or AB tele-health*  OR TI telemed* or AB telemed*  OR TI tele-med* or AB tele-med*  OR TI ehealth* or AB ehealth*  OR TI e-health* or AB e-health*  OR TI electronic consult* or AB electronic consult*  OR TI econsult* or AB econsult*  OR TI remote consult* or AB remote consult*  OR TI Virtual Consult* or AB Virtual Consult*)  AND  3.(TX video  OR TX video conference  OR TI Skype OR AB Skype  OR TI web-camera OR AB web-camera  OR TI webcam OR AB webcam  OR TI web-cam OR AB web-cam  OR TI Facetime OR AB Facetime)  AND  4. EM 201901-202003 |
| --- | --- |

Web of Science

| Bottom-up | 1. (((TI=scale OR TS=scale AND (TI=bring* OR TS=bring* OR TI=brought OR TS=brought OR TI=taking OR TS=taking OR TI=increas* OR TS=increas* OR TI=going OR TS=going OR TI=implement OR TS=implement)) OR (TI=spread OR TS=spread AND (TI=innovation* OR TS=innovation* OR TI=intervention* or TS=intervention* OR TI=technolog* OR TS=technolog* OR TI=practice OR TS=practice OR TI=care OR TS=care)) OR TI=scale up OR TS=scale-up OR TI=scaling up OR TS=scaling up OR TI=scalability OR TS=scalability OR TI=implementation OR TS=implementation)  AND  2. (TI=telehealth* OR TS=telehealth* OR TI=tele-health* OR TS=tele-health* OR TI=telemed* OR TS=telemed* OR TI=tele-med* OR TS=tele-med* OR TI=ehealth* OR TS=ehealth* OR TI=e-health* OR TS=e-health* OR TI=electronic consult* OR TS=electronic consult* OR TI=econsult* OR TS=econsult* OR TI=remote consult* OR TS=remote consult* OR TI=Virtual Consult* OR TS=Virtual Consult*)  AND  3. (TS=video OR TS=video conference OR TI=Skype OR TS=Skype OR TI=web-camera OR TS=web-camera OR TI=webcam OR TS=webcam OR TI=web-cam OR TS=web-cam OR TI=Facetime OR TS=Facetime)  AND  4.PY=(2019-2020))  AND  5.LANGUAGE: (English) |
| --- | --- |
