# Supplementary material for: Spread, Scale-up, and Sustainability of Video Consulting in Health Care: Systematic Review and Synthesis Guided by the NASSS Framework
Source: J Med Internet Res. 2021 Jan 26;23(1):e23775. doi: 10.2196/23775 (PMC7837451; doi:10.2196/23775)
Supplement: Multimedia Appendix 3 [file jmir_v23i1e23775_app3.docx]

**Multimedia Appendix 3**. Summary of papers included in the review.

| **Paper and funding** | **Setting** | **Clinical focus** | **Definition of telehealth** | **Service model and technology** | **Theoretical framework** | **Research design and data collected** | **Degree of spread attempted** | **Challenges and opportunities for spread and scale-up** |
| --- | --- | --- | --- | --- | --- | --- | --- | --- |
| Brooks et al (2012) [33], National Centre on Minority Health and Health Disparities | Veterans Affairs telemental health clinics, United States | Individual and group therapy, medication management, and diagnostic assessment for American Indian veterans with post-traumatic stress disorder | None given | Patients at rural reserve sites connected to mental health care at a large health center via a video link, facilitated by a telehealth outreach worker | Diffusion of Innovation [44] | Telephone interviews with professionals (n=39; 80% male, 56% white, 57% graduates or medics) to understand the facilitators and challenges of shifting from initial implementation to local spread. Data collection dates not reported. | Diffusion of three telemental health clinics | Two fully operational telehealth clinics and one in early stages, enabled by recognition of need for posttraumatic stress disorder treatment in the community, interorganizational cooperation, patient and staff acceptance, and presence of telehealth outreach worker. Challenges included interorganizational cooperation and communication, cultural sensitivity, and transport (of veterans to rural telehealth clinics). |
| Latifi et al (2014) [41], International Trust Fund, Ministry of Foreign Affairs of Republic of Slovenia | Specialist care, Cabo Verde | Routine specialist care for all citizens | Focused on “telemedicine carts,” combining videoconferencing capability with integrated peripherals to support wide-ranging clinical activities | Nationwide implementation of telemedicine carts and videoconferencing equipment (using bespoke software created by implementing organization) to connect remote centers with specialists, combined with continuing medical education and a library platform | Initiate-build-operate-transfer (IBOT) [45] | Prospective study of widespread implementation using descriptive data (number of video consultations/referring providers), plus detailed analysis of nationwide implementation of video consultation technology. Data collected November 2011 to December 2013. | Nationwide spread of 10 telemedicine centers on 9 islands over 3 years | Nationwide spread enabled by national prioritization, government cooperation and ongoing support and training. Initial steady increase in the number of teleconsultations (in the first 7 months), with a greater number of consultations in centers with more specialties and among clinicians who had trained abroad. Challenges included presence of telehealth champions, internet connectivity, training, and technical support. |
| Darkins et al (2014) [43], funding not reported | Specialist and primary care, Veterans Health Authority, United States | Primary and specialist care for US veterans | “Electronic information and telecommunication technologies to support clinical healthcare, education, public health, and administration; making use of video conferencing, store-and-forward imaging, streaming media, terrestrial and wireless communications” [46] | Use of information and telecommunication technologies, including video consultation technologies, to deliver consultations and care over a 20-year period; further details of the technology not reported. | Diffusion of Innovation [44] | Retrospective case analysis (1994-2014) of telehealth development over 20 years, involving documentation review, discussions with stakeholders, and author reflections. Participant characteristics not reported. | A 20-year program following initial implementation to spread to 152 medical centers and establishment of a national telemental health center | Widespread growth of telehealth, including 2000-fold increase in clinical video telehealth contacts over 20 years. Widespread implementation enabled by a favorable organizational mindset, strong evidence base, telehealth support services, specific telehealth training centers, fewer logistical/reimbursement challenges with Veterans Affairs, and clinician buy-in. |
| Lindsay et al (2015) [40], Houston Veterans Affairs | Five Veterans Affairs medical centers and their associated community clinics, United States | Psychotherapy for posttraumatic stress disorder for US veterans | None given | Establishment of evidence-based practice for posttraumatic stress disorder via video telehealth over a 2-year period; details of the technology not reported | Promoting Action on Research Implementation in Health Services (PARIHS) [47] | Prospective implementation study (January 2012 to September 2013) piloting external facilitation as an approach to expanding access to psychotherapy in the community. Statistically measuring increase in psychotherapy sessions via video telehealth, primarily between sites participating in the facilitation intervention and those not participating. | Five Veterans Affairs medical centers and associated community clinics | A total of 27 clinics were established in 2 years, with a 46-fold increase in the number of video-telehealth contacts across five Veterans Affairs centers. Growth facilitated by external facilitation (including substantial training), leadership engagement, and custom site-specific implementation plans. Poststudy spread included 2 more community clinics. |
| Bhatta et al (2015) [32], funding not reported | Three rural telemedicine clinics covering specialist and primary care, Nepal | Specialist care for rural residents in Nepal | Medical activities involving distance, and covering diagnosis, clinical management, treatment, and education; involving distance for health care workers and patients [48] | Rural telemedicine program started by the Government of Nepal two years prior to the study in 30 remote hospitals to provide specialist care to rural areas; details of the technology not reported | No a priori theoretical or implementation framework but a mention of analysis of the sociotechnical aspects of rural telemedicine | Interpretative case study (December 2012 to January 2014) involving informants (n=15) who were policy makers and program implementers in the rural telemedicine programs. Demographic information not reported. | 30 remote district hospitals (three of which were included in the study) | Gradual expansion of video conferencing at 3 hospital sites, enabled by need for specialist care. Challenges included internet connectivity, English-language skills (to use the technology), cultural difficulties, inconsistent staffing, and lack of sustainable funding or government support. |
| Wade et al (2016) [39], Australian Government | Adelaide, Australia | Rehabilitation, palliative, and geriatric outreach services | None given | Pilot of home telehealth (using Vidyo, plus multiple peripherals) [49] for specialist outreach already in place for 18 months, with focus shifting to plan for scale-up of clinical activities covering diagnosis, management, treatment, and education | Grounded theory [50] | Action-based research study [51] involving semistructured interviews (n=19) with senior clinicians, health service managers, and policy makers, and a subsequent deliberative forum to select the preferred implementation approach and management model. Dates and demographic information not reported | Large-scale implementation of home telehealth in South Australia | Limited scale-up, largely planning. Stakeholders approached scale-up with a collaborative consortium model combined with evidence-based change management. Plans for widespread implementation focused on health service leadership support to overcome potential challenges like lack of funding and resistance to change by clinicians. |
| Alami et al (2017) [36], Laval University, Canada (scholarship) | Specialist and primary care, Norway | All telemedicine use in Norway over a 7-year period | Use of communications networks for delivering health care services and medical education from one geographical location to another [52] | Use of videoconferencing nationally from 2009-2015 to conduct a consultation or examination, establish a diagnosis, or provide treatment at distance; details of the technology not reported | No a priori theoretical or implementation framework | Mixed methods approach using qualitative interviews (n=9) with stakeholders at national, regional, and, local levels, and quantitative data from the Norwegian Patient Registry (2009-2015). Demographic information not reported | National spread across 4 health regions and 29 hospitals | Spread of telemedicine low and fragmented across discrete organizations. Lack of spread and uptake reported as due to a fragmented health system resulting in unclear telemedicine management, governance, security, and information management. High clinician turnover, resistance to organizational change, and reimbursement of video conferencing for general practitioners were major obstacles. |
| Greenhalgh et al (2018) [38] and Shaw et al (2018) [2]^a^, National Institute for Health Research | Three outpatient clinics in a large inner-city NHS Trust, United Kingdom | Routine outpatient appointments for young adults with diabetes, antenatal diabetes, and postoperative follow-up for cancer surgery | Remote video consultations VoIP | Video consulting services using Skype, involving clinicians and patients, following 4 years of prior planning and implementation | Nonadoption, Abandonment, and challenges to the Scale-up, Spread, and Sustainability (NASSS) framework [23] | Qualitative, mixed method, and multilevel study (2015-2017) focused on clinician-patient video consultations (18 for diabetes, 12 for cancer), organizational implementation, and national-level policy; involved 24 staff (9 clinicians, 5 managers, 3 support staff, and 7 administrators), 50 patients, and 36 national-level stakeholders | Spread to 3 clinical settings within a large, multisite NHS hospital over 2 years | Steady progress, with 3%-20% of outpatient appointments routinely shifted to video in 2 years, enabled by patient support in selected services, clinical champions, high-level organizational support, appropriate technology, and clear benefits for all stakeholders. Challenges included information governance, technical setup, and redesign of existing clinical pathways. |
| Bauer et al (2018) [37], Department of Veterans Affairs Office of Telehealth Services | Medical centers and community clinics within the Veterans Health Administration care system, United States | Routine psychotherapy for bipolar disorder | Clinical video technology (CVT), a real-time videoconferencing technology used to provide health care services | Videoconferencing-based chronic care model for bipolar disorder, implemented 5 years prior to this evaluation; details of the technology not reported | Integrated PARIHS (i-PARIHS) [53] (qualitative) and Reach, Efficacy, Adoption, Implementation, and Maintenance (RE-AIM) [54] (quantitative) | Mixed method quality improvement and program analysis (November 2011 to May 2016) combining quantitative data with interviews to examine program growth, usefulness, and challenges and opportunities to implementation and sustainability. | Spread from 0 to 35 sites nationwide | Clinical video technology established in 35 distinct sites across 12 US states and 8 (of 20) Veterans Affairs regions over 5 years. Scale-up and spread were enabled by choice of technology, support for implementation into existing workflows, and strong infrastructure to support implementation. Site-level challenges varied, but included loss of telehealth support staff, lack of provider buy-in, availability of telehealth equipment, and the intensity of scheduling. |
| Interian et al (2018) [34], Veterans Affairs, Quality Enhancement Research Initiative | Veterans Health Administration (VHA) facilities with varying levels of video-to-home visits, United States | Psychotherapy for diverse mental health issues, delivered to rural, suburban, and urban patients | Video-to-home (V2H) telehealth, allowing patients to connect with a provider from their own homes by using a computer webcam. | For video-to-home telehealth, "patients connect with a provider from their own homes by using a computer Webcam"; implemented 5 years prior to this evaluation. | Consolidated Framework for Implementation Research (CFIR) [55] | Quantitative activity data (2014-2015), combined with semi-structured interviews (n=33; 28 direct providers and 5 nonproviders) with mental health providers at 3 Veterans Health Administration facilities who were familiar with, had experience with, or had never used video-to-home visits. Demographic information not reported. | Three Veterans Health Administration facilities | Local spread from 3%-53% over one year in 3 Veterans Health Administration facilities. Facilitators included provider-facing support for/promotion of telehealth, site champions, and logistical support. Growth was slowed by a lack of provider buy-in and lack of experience with video-to-home visits, concerns about the clinical compatibility of the service, and requirement of logistical support to support provider interest. |
| Martinez et al (2017) [35], Department of Veterans Affairs | Veterans Health Association medical centers, United States | Spinal cord Injuries and disorders | Clinical video telehealth (CVT), a real-time videoconferencing technology used to provide health care services | Clinical video telehealth to connect patients receiving care at local care facilities with providers at the Veterans Health Administration centers delivering spinal cord injury and disorder services; details of the technology not reported | Sittig and Singh’s sociotechnical framework [56] | Qualitative evaluation (January to July 2014) of social and technical factors influencing implementation using semistructured stakeholder interviews (n=40; 77.5% female, 42.5% aged 46-55 years) from 33 different Veterans Health Administration centers | Not reported | Extent of scale-up not reported, while 33/69 sites reported that growth was enabled by on-site champions, senior support, trusted relationships with the teams at both “ends,” and strong communication. Challenges included complex implementation (in light of people/processes required), as well as availability of adequate resources, appropriate equipment, space, personnel, technology, and support. |
| Lindsay et al (2019) [42], Veterans Affairs, Office of Rural Health | Houston Veterans Affairs Medical Center, United States | Mental health care | Video telehealth to home (VTH), which enables patients to connect with a mental health provider directly from home or another location | Full integration of video-to-home mental health care over a 5-year period (leading to a browser-based “VA Video Connect” program); patients connect remotely to a provider via their own device | Personalized Implementation for Video Telehealth (an implementation facilitation approach) [57] | Prospective implementation study (2013-2018) as a clinical demonstration project and outcome evaluation including quantitative and qualitative data collection. Demographic information not reported. | Spread across a Veterans Affairs medical center | Over 6 years, there were 6 times the number of users and 6.5 times greater frequency of consults (compared to the national median). Widespread and sustained growth enabled by perceived clinical compatibility for low-incidence mental health issues and the occurrence of a natural disaster (Hurricane Harvey) necessitating remote contact. Strong leadership, external facilitation for implementation, starting small/being flexible, logistical support, investment of resources, organizational preparedness, and ongoing assessment of the technology aided spread and scale-up. |

^a^This study was reported in two separate articles (one journal paper and one report to funders), hence both are included in the same row.
